# Una aplicación App para micetismos en urgencias

**DOI:** 10.1515/almed-2020-0013

**Published:** 2020-04-07

**Authors:** Salvador Ventura Pedret, Esther Solé Llop, Josep M. Queraltó Compañó, Jaume-Miquel March-Amengual

**Affiliations:** Laboratori Clínic Metropolitana Sud, Institut Català de la Salut, Comisión de Toxicología y Monitorización de Fármacos de la SEQC^ML^ , Barcelona, España; Hospital Clínico “Lozano Blesa” de Zaragoza, Salud Aragón, Comisión de Toxicología y Monitorización de Fármacos de la SEQC^ML^ , Zaragoza, España; Comisión de Toxicología y Monitorización de Fármacos de la SEQC^ML^ , Barcelona, España; Grupo de investigación en Metodología, Métodos, Modelos y Resultados de Salud y Ciencias Sociales (M3O), Facultad de Ciencias de la Salud y Bienestar, Centro de Investigación en Salud y Asistencia Social (CESS), Universidad de Vic, Universidad Central de Cataluña (UVIC-UCC), Vic, España

**Keywords:** aplicacion App, intoxicaciones, micetismos

Estimado Editor,

En la década de 1980, se produjo en Alemania una intoxicación por ingestión del hongo *Galerina sulciceps*, una de las especies más tóxicas del planeta. En caso de ingesta, la concentración en anatoxinas produce una mortalidad del 75%, en comparación con el 30% de la mortalidad estimada en la intoxicación sin tratamiento por *Amanita phaloides*. Llama la atención el hecho de que *G. sulciceps* es un hongo originario de Java y Ceilán. Sin embargo, no es un fenómeno aislado ya que en noviembre de 2013 se registraron dos intoxicaciones por *G. sulciceps* en la ciudad de Duyun, provincia de Guizhou, China [1] lo que sugiere su expansión progresiva en el planeta. El *Clitocybe amoenolens* produce el síndrome acromelàlgico causado por el ácido acromélico por afectación del metabolismo del triptófano en las fibras mielinizadas del sistema nervioso autónomo. Dependiendo de la dosis, se manifiesta como edema y enrojecimiento de dedos de manos y pies y dolores paroxísticos muy intensos de aparición nocturna. Sorprende que esta especie, descubierta inicialmente en el Atlas marroquí, se ha encontrado también en la Alta Saboya, Francia [2], en Almarza de Cameros, La Rioja, y en la provincia de Guadalajara [3] e incluso se ha descrito su presencia en Italia [4]. Aún más preocupante es la extensión de intoxicaciones por *Podostroma cornu-danae*. Esta especie puede confundirse con ejemplares jóvenes de *Ganoderma lucidum*, presente en los bosques peninsulares donde por su forma es conocida como “pipa”. No es comestible, pero en Extremo Oriente se consume en infusión e incluso se le ha atribuido propiedades anticancerosas, aunque con poca evidencia científica [5], [6]. Pero sucede que *Podostroma* es responsable de una intoxicación característica y grave, causada por micotoxinas de la familia de los tricotecenos: satratoxina, verrocarina y roidrina. Estas toxinas inhiben la síntesis de proteínas en los ribosomas al bloquear la peptidil transferasa. Descamación de la piel, cansancio, pancitopenia y fallo renal constituyen la clínica habitual. La mortalidad entre los afectados es alta y parece ser que en 2018 se produjeron cuatro casos de intoxicación en Europa [7]. Otros ejemplos de hongos invasores son la *Amanita smithiana* en Baleares y la *Macrolepiota venenata* (*Lepiota morganii*) importada en cepellones de abetos nórdicos a finales del siglo XX [8].Por último, cabe señalar que antes de 1949, en Argentina, no había constancia de *Amanita phalloides* [9].

Estas anécdotas pretenden llamar la atención sobre cómo el fenómeno de la globalización afecta también a las intoxicaciones causadas por hongos. La facilidad en el transporte de mercancías ha hecho que estas especies se hayan diseminado a través de la importación de plantas o maderas gracias a condiciones climatológicas favorables para su propagación, a la ausencia de competidores y a la semejanza engañosa con especies autóctonas comestibles. Este fenómeno augura un aumento de los micetismos en el futuro.

Una de las características de estas intoxicaciones es la demora en el diagnóstico. Si a esto se añade el probable incremento de síndromes importados y el gran desconocimiento de los mismos, puede hacer que sea necesario y conveniente disponer de una herramienta que facilite el diagnóstico precoz y la instauración rápida del tratamiento más apropiado.

Los recursos que ofrecen las tecnologías de la información y comunicación (TIC) en el ámbito sanitario son especialmente útiles en aquellas áreas que requieren una acción inmediata, como en medicina de urgencias. Para fines profesionales, WhatsApp es una aplicación que utiliza un 31% de la población de Estados Unidos según el informe realizado en 2016 por el *Interactive Advertising Bureau* sobre el uso de redes sociales [10] donde se indica que, en Estados Unidos, el 84% de los médicos posee un Smartphone, el 38% una iPad y que el 59% lo utiliza en su práctica clínica. España, con 30,5 millones de usuarios de WhatsApp, es el noveno país del mundo, solo por debajo de Alemania e Italia, según un estudio de eMarketer de agosto de 2019 (disponible en https://www.emarketer.com/newsroom/index.php/whatsapp-a-key-driver-of-mobile-messaging-growth/). Como consecuencia, el uso del correo electrónico ha disminuido en beneficio de aplicaciones móviles [11].

En este contexto, la Comisión de Toxicología y Monitorización de Fármacos de la SEQC^ML^, en colaboración con el Departamento de Ingeniería de la Universitat de Vic, diseñó una aplicación web de uso en dispositivos móviles, MicoApp, como ayuda para el diagnóstico precoz de las intoxicaciones por micetismos para uso del personal sanitario [12]. Se trata de una aplicación de funcionamiento intuitivo, accesible gratuitamente a través de la página web http://www.micoapp.uvic.cat, mediante un registro de usuario. Se indica explícitamente que es una herramienta “dirigida a personal sanitario” y “no substituye a ningún criterio diagnóstico que pueda ser emitido por el facultativo consultante, siendo una herramienta de ayuda y soporte al diagnóstico". El funcionamiento y características técnicas de esta aplicación se pueden encontrar en [12].

El diagnostico a tiempo es crucial en cualquier tipo de intoxicación. Sin embargo, solo un 50% de los pacientes acude a urgencias siempre y cuando los síntomas son lo suficientemente alarmantes. Lamentablemente esto se traduce en una demora en el diagnóstico con las obvias consecuencias médicas. MicoApp puede resultar útil como elemento de consulta, pero también como instrumento docente ya que con él es posible recrear muchas situaciones clínicas, incluyendo intoxicaciones mixtas por diferentes hongos, de modo que en una situación real pueda realizarse con mayor facilidad el diagnóstico adecuado en el menor tiempo posible. Hay que tener en cuenta que el aprendizaje progresivo en un contexto de situaciones simuladas tiene cada vez más adeptos en el campo docente. El hecho que esté abierto a incorporar, en futuras versiones, elementos de inteligencia artificial como redes neuronales, lo hacen especialmente atractivo para la docencia. Así mismo, en futuras versiones podrían incluirse intoxicaciones por drogas de abuso, sustancias de origen vegetal o animal, fármacos, etc.

Sin embargo, es preciso recalcar que MicoApp no supone un procedimiento diagnóstico alternativo o sustitutivo de metodologías que ofrecen resultados definitivos [13], [14].
